# Stereotactic Body Radiation Therapy for Prostate Cancer: Review of Experience of a Multicenter Phase I/II Dose-Escalation Study

**DOI:** 10.3389/fonc.2014.00319

**Published:** 2014-11-26

**Authors:** D. W. Nathan Kim, Christopher Straka, L. Chinsoo Cho, Robert D. Timmerman

**Affiliations:** ^1^Department of Radiation Oncology, University of Texas Southwestern Medical Center, Dallas, TX, USA; ^2^Department of Radiation Oncology, University of Minnesota, Minneapolis, MN, USA

**Keywords:** SBRT, prostate cancer, rectal toxicity, low risk, intermediate risk

## Abstract

**Introduction:** Stereotactic body radiation therapy (SBRT) is an area of active investigation for treatment of prostate cancer. In our phase I dose-escalation study, maximum-tolerated dose (MTD) was not reached, and subsequently phase II study has been completed. The purpose of this article is to review our experiences of dose-escalated SBRT for localized prostate cancer.

**Methods and materials:** Patients enrolled to phase I/II study from 2006 to 2011 were reviewed. Prescription dose groups were 45, 47.5, and 50 Gray (Gy) in five fractions over 2.5 weeks. Toxicity and quality of life questionnaire data were collected and analyzed. Descriptive statistics were obtained in the form of means, medians, and ranges for the continuous variables, and frequencies and percentages for the categoric variables.

**Results:** Ninety-one patients were enrolled from five institutions. Median follow-up for prostate specific antigen (PSA) evaluation was 42 months. PSA control remains at 99%. While the MTD was not reached in the phase I study, excess high grade rectal toxicity (10.6%) was noted in the phase II study. The 13 patients treated to 50 Gy in the phase I study that did not have high grade rectal toxicity, in retrospect met these parameters and have not had further events on longer follow-up.

**Conclusion:** Prostate specific antigen control rate, even for patients with intermediate risk, is thus far excellent at these dose levels. This study provides a platform for exploration of SBRT based clinical trials aimed at optimizing outcome for intermediate and high risk patients. High grade toxicities specifically related to the rectum were observed in a small but meaningful minority at the highest dose level. Dose constraints based on physiologic parameters have been defined to mitigate this risk, and strategies to minimize rectal exposure to such doses are being explored.

## Introduction

Stereotactic body radiation therapy (SBRT)/Stereotactic ablative radiotherapy (SABR) remains an area of active investigation for prostate cancer research. Most recently, we have completed a multi-institutional phase I and II study of SBRT for low and intermediate risk prostate cancer patients (1, 2). Our study was unique on several fronts. First of all, it was a multi-institutional design, and patients were enrolled from five different institutions using a broad variety of radiation delivery platforms. Secondly, it involved a prospective dose-escalation phase I study design that enrolled at least 15 patients per dose level. Third, this study involved use of doses that are significantly higher than what has been typically reported for use in the retrospective prostate SBRT literature. Finally, our experience of an unexpected change in toxicity profile from phase I to the phase II findings, highlights the importance of conducting a prospectively designed phase II study to better assess the efficacy and safety of what is determined to be the maximum-tolerated dose (MTD) from a limited number of patients in the phase I efforts (2). In this article, we will briefly update readers of our experiences, including informing readers of potential for rectal toxicity when considering use of ablative radiation doses for tumors near bowel structures, and discuss potential implications when conducting future clinical trial design of high-dose SBRT for prostate cancer.

## Materials and Methods

### Patients and eligibility

From 2006 to 2011, 91 patients were enrolled to an institutional review board (IRB) – approved multi-institutional phase I/II clinical study, and analyzed. At the time of study design, it was determined that this would be a phase I/II study, and therefore pooling of data between the two studies was anticipated. This study was registered with National Cancer Institute, and clinicalTrials.gov identifier is NCT00547339. Please see patient eligibility section in prior publication (1). The only change from eligibility criteria in the phase II component was exclusion of patients who are actively immune-suppressed due to safety concerns (1).

### Statistical design

#### Phase I study design

This study was designed as a prospective dose-escalation study. The goal was to escalate the dose of five-fraction SBRT to the MTD or 50 Gray (Gy). Dose-limiting toxicity (DLT) was defined as grade 3–5 gastrointestinal (GI), genitourinary (GU), sexual, or neurologic toxicity attributed to therapy occurring within 90 days of registration using Common Terminology Criteria of Adverse Events version 3 (CTCAE v3.0). Escalation was allowed to occur if four or fewer patients within a cohort of 15 patients experienced DLT within 90 days of follow-up at a given dose level. This escalation rule is the same as the traditional 3 + 3 design in which <33% DLT rate at the current dose level leads to further dose escalation. Using a larger number of patients per dose level is justified for this trial on the basis of the previously referenced SBRT and high-dose rate (HDR) experiences that predict efficacy even with the starting dose. As with the traditional 3 + 3 design, sequential enrollment served as a protection to limit potential overdosing. Furthermore, 15 patients at each level allowed us to more accurately estimate the DLT rate and to study other end points related to the enrolled patients. The MTD was defined as the dose level immediately below the intolerable dose.

#### Toxicity stopping rules for phase II component

Three interim analyses of toxicity were planned after 25% (12 patients), 50% (24 patients), and 75% (36 patients) of the total number of evaluable patients to be accrued in phase II. These interim analyses were done after patients finished their toxicity assessment periods for each group (i.e., 90 days of post therapy follow-up). The following early stopping rules reject the null hypothesis that the toxicity rate is ≤10% in favor of the alternative hypothesis that the toxicity rate is at least 30% with an overall Type I error rate of no more than 0.05:

six or more cases of unacceptable toxicities out of the first 12 evaluable patients, or
seven or more cases of unacceptable toxicities out of the first 24 evaluable patients, or
eight or more cases of unacceptable toxicities out of the first 36 evaluable patients.

The final analysis tested the same null hypothesis using the rejection rule of 10 or more patients with unacceptable toxicities out of the total sample of 47 evaluable patients. This insured an overall significance level of 0.05 for the final conclusion. After 47 of the 50 accrued patients were evaluable, then the first 47 evaluable patients were used for this analysis. If the number of unacceptable toxicities observed demonstrate via the monitoring rules above that the treatment-related unacceptable toxicity rate is 30% or more, consideration was made for stopping the study. In this case, the study chair, study principal investigators (PIs), and statistician reviewed the toxicity data along with the Data Safety Monitoring Committee (DSMC) and made appropriate recommendations about continuing the study. Additionally, the treatment-related unacceptable toxicity rates were monitored during the 5-year follow-up period. If the unacceptable toxicity rate exceeded 30% at any time during the 5-year follow-up period, the study chair, study PIs, and statistician would review the toxicity data along with the DSMC and make appropriate recommendations about reporting the information.

In the phase II study, all patients were treated with 10 Gy per fraction to a total dose of 50 Gy. Otherwise, planning and treatment was described previously (1).

#### Study end points and statistics

##### Study end points

In the phase I study, the primary endpoint was to determine MTD. Secondary end points were late toxicity (occurring 90 days from treatment), patient-reported toxicity/Quality of Life (QOL), and prostate specific antigen (PSA) response. The Expanded Prostate Cancer Index Composite (EPIC) questionnaire and American Urological Association (AUA) symptom scores were collected at baseline and at 1.5, 3, 12, and 18 months after treatment. Patients were observed with PSA, history, and physical examination every 3 months for the first year, every 6 months for years two to three, and yearly starting 4 years after treatment. The nadir +2 ng/mL failure definition was used for biochemical control.

The phase II component of this study was designed to test whether late GU/GI toxicity is above 10%. The sample size for phase II was determined so that the probability of rejecting the treatment because of excessive late toxicity was 90% if the true late toxicity rate is 23% or higher. Assuming an exponential distribution for time from the end of the acute period (270 days) to the occurrence of late toxicity, the hazard rate for the expected 10% toxicity rate, and the unacceptable 23% toxicity rate was 0.006/month and 0.015/month, respectively. Following the asymptotic property of the observed hazard and using *Z*-test for the logarithm of the hazard ratio, 12 cases with severe late GU/GI toxicity would be required. Thus, 47 patients were required to be accrued within 3–4 years and be followed for 270 days after the acute period (i.e., a total of 540 days) to have a statistical power of 90% with a one-sided significance level of 0.05. Considering 5% ineligible cases and lack-of-data cases, the sample size of the phase II component of this study was planned to be 50 patients. With the data for the 47 patients from Phase II trial plus an additional 44 patients from the previously conducted Phase I trial, our total sample size in this analysis is 91. Patients from both trials were recruited from multiple institutions.

Descriptive statistics were obtained in the form of means, medians, and ranges for the continuous variables, and frequencies and percentages for the categorical variables. All statistical analyses were performed at the 0.05 significance level using SAS 9.2 for Windows (SAS Institute Inc., Cary, NC, USA).

### Planning and treatment

#### Planning, setup, and dose parameters for the clinical trial

Fiducial markers consisting of gold seeds (Calypso beacons were permitted) were placed within the prostate approximately 1 week before radiation simulation. A bowel regimen consisting of 30 mL of milk of magnesia the evening before and a Fleet enema 30–60 min before simulation and each treatment was used, along with the insertion of a 60–100 cm^3^ rectal balloon. Patients were instructed to have a full bladder for simulation and treatment. A thin, flexible catheter was used to delineate the urethra at simulation only. Magnetic resonance imaging (MRI) and/or computed tomography (CT) was used to define the prostate and organs at risk. The prostate was expanded uniformly by 2–3 mm to create the planning target volume (PTV) based on institutional PTV guidelines. SBRT was delivered via ring gantry helical accelerator (Tomotherapy; TomoTherapy Inc., Madison, WI, USA) or on a linear accelerator with image guidance (Trilogy; Varian Medical Systems, Palo Alto, CA, USA and Synergy; Elekta AB, Stockholm, Sweden) with energies of 6–15 MV. None of the centers used a Cyberknife platform to treat enrolled patients, mostly related to availability or arbitrary preferences. Intensity modulated radiotherapy (IMRT) was utilized in all cases. The dose was prescribed to cover 95% of the PTV. Rapid dose fall-off outside the PTV was prioritized over PTV dose uniformity, resulting in considerable dose heterogeneity within the PTV. Tissue heterogeneity correction was used in all cases. A rectal balloon was used, which helped push the lateral and posterior walls away from the high-dose region, which at the time of trial design, was felt to be advantageous for sparing stem cells whose migration would be required to help heal the anterior rectal wall after radiation therapy. The rectal wall was divided and separately contoured into anterior, lateral, and posterior walls in the region of the PTV. The anterior wall was allowed to receive no more than 105% of the prescription dose. No more than 3 cm^3^ of the lateral walls were allowed to receive 90% of the prescription dose. The posterior rectal wall maximum dose was limited to 45% of the prescription dose. The bladder wall (outer 5 mm of the entire bladder contour) was limited to 105% of the prescription dose with no more than 10 cm^3^ receiving 18.3 Gy or greater. The maximum prostatic urethra dose was limited to 105% of the prescription dose.

### Toxicity

Toxicity events and ongoing cumulative toxicity reports from all institutions were reviewed at regular intervals by the Simmons Comprehensive Cancer Center DSMC and an independent medical monitor with prostate cancer expertise. Common Terminology Criteria of Adverse Events (CTCAE) version 3.0 was used. Acute and delayed toxicity were defined *a priori* on the clinical study protocol, as toxicity occurring <270 days and occurring or persisting ≥270 days from start of protocol treatment, respectively. If the number of unacceptable toxicities observed demonstrate via the monitoring rules that the treatment-related unacceptable toxicity rate is 30% or more, consideration was made for stopping the study. The study chair, PIs, and statistician would review the toxicity data along with the DSMC to make appropriate recommendations about continuing the study.

## Results

### Patient characteristics

Total of 91 patients were eligible for analysis for this study, including 44 in the phase I study, and 47 in the phase II study. Initially 45 patients were enrolled in the phase I study, but 1 patient was deemed ineligible upon re-review of his pathology demonstrating Gleason 9 features. This patient was omitted from further analysis. Complete list of patient characteristics for phase I and II studies have previously been published (1, 2). Our study enrolled from three institutions during the phase I study, and this was expanded to five institutions during the phase II study. As seen in Table 1, of the five institutions, four are academic centers, and one is a community practice. While majority of the patients (64%) for the two studies were enrolled from the PIs institution (UT Southwestern, Dallas, TX, USA), a significant number of patients enrolled in the two studies (36%) came from the other four institutions. The study did permit multiple platforms for treatment modality to be used, with three of the five centers using Tomotherapy platform, while the other two used standard linear accelerator technology to perform the SBRT.

**Table 1:** **Patient enrollment by institutions**.

Institution	45 Gy	47.5 Gy	50 Gy	Phase II	Total	Treatment platform used
University of Texas Southwestern Medical Center, Dallas, TX, USA	14	8	9	27	58	LINAC
University of Minnesota, Minneapolis, MN, USA	1	4	4	11	20	Tomotherapy
Prairie Lakes Hospital, Watertown, SD, USA		3	1		4	Tomotherapy
University of Colorado, Denver, CO, USA				7	7	LINAC
University of Florida Health Cancer Center-Orlando (previously known as MD Anderson – Orlando)				2	2	Tomotherapy
Total	15	15	14	47	91	

### PSA control

**Figure 1:**
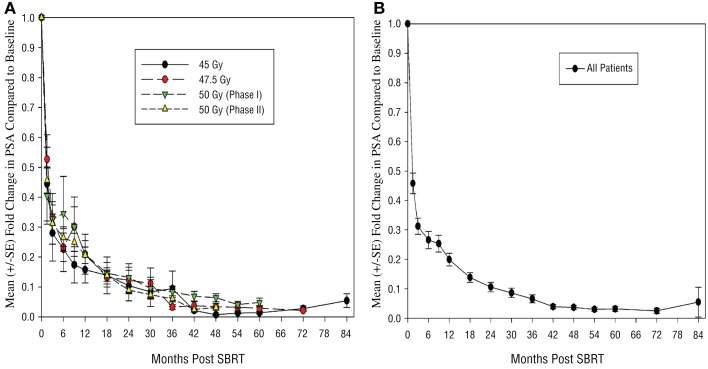
**Plot of mean fold change in PSA compared to baseline levels (A) by individual dose groups and (B) for all patients**. S.E., standard error; PSA, prostate specific antigen; Gy, gray.

Median f/u for PSA evaluation for all patients was 42 months (m) [range (r) 1.5–84 months]. For phase I study, median f/u for PSA evaluation was 54 months (r 3–84 m). For phase II study, median f/u for PSA evaluation was 36 m (r 1.5–60 m). Median f/u for PSA evaluation for patients treated to 45 Gy was 60 m (r 3–84 m), to 47.5 Gy was 54 m (r 30–72 m), and to 50 Gy (phase I study) was 54 m (r 3–60 m). PSA control rate to date is 99% as only 1 patient (out of 91) has demonstrated failure to therapy. This patient has Gleason score (GS) 4 + 3 (<50% core involvement), pretreatment PSA 7.4, and cT2b intermediate risk disease (or unfavorable intermediate risk by Memorial Sloan Kettering criteria). This patient was treated on the 45 Gy arm of the phase I study. On evaluation, he was found to have regional (imaging and biopsy proven external iliac nodal failure) 41 months from time of initiation of SBRT. He subsequently developed L3 bone metastases 45.5 months post initiation of therapy. At the time of biochemical failure, he also underwent prostate biopsy, which demonstrated two microscopic foci of residual prostatic adenocarcinoma at the right base, which was too miniscule to be accurately given a Gleason grade. He was eventually started on salvage systemic therapy 46 months post SBRT. If we consider only the 9 Gy × 5 fraction group, PSA control rate in this group is 93.3% with a median f/u of 60 months. If we consider by Gleason grade, PSA control is 100% in patients with GS 6 or 3 + 4 disease, and 93.3% in GS 4 + 3 patients. Figure 1A illustrates the mean PSA trend changes for patients treated in the different arms of the study showing no obvious difference in PSA trends between these groups. Figure 1B demonstrates PSA trends for all patients treated on this study. As can be seen, on the average, patients had >50% reduction from their baseline PSA values within first 6 months, followed by steady decline over the next 3 years.

### Rectal toxicity in the 50 Gy group: Phase I vs. phase II experience

We have recently reported on our rectal toxicity outcome from the phase I/II study (2). What is most remarkable from our experiences was that our toxicity profile was markedly different in the phase II experience despite having 15 patients treated at each dose level in the phase I study (which is far larger than typical number of patients treated per dose level for phase I drug trials). As seen in Table 2, only 1 out of 14 patients developed grade 3+ rectal toxicity in the phase I study. As discussed previously, this patient was on immunosuppressants due to history of kidney transplantation. Given that only one patient developed serious rectal toxicity in all of the phase I study groups, there was strong suspicion that this event may have been related to the chronic immune-suppressed state of this patient. Regardless, MTD was not reached in the phase I study, and a decision was made to proceed to the phase II study using this dose, with the exclusion of patients on immunosuppression (1). However, in the phase II study, the toxicity profile changed, and five patients (10.6%) developed high grade rectal toxicity at the time of data analysis with a median follow-up of 24.5 months. Of these total six patients, five had issues involving the anterior rectum, all of whom underwent a diverting colostomy, and for details, we refer readers to our recent manuscript’s Table 3, which details these issues (2). Of note, one of these patients had a posterior Dieulafoy lesion, which was cauterized and resolved next day. While we did not feel that this case was related to the SBRT, but as the patient did require hospitalization, was documented as a high grade rectal event in our study (2). While we await full reporting of toxicity data from the next interim analysis with longer follow-up, we are as of yet aware of only one additional patient that has developed high grade delayed rectal toxicity within the phase II group (12.8%), and the full data from the phase II experience will be reported in future publications. Furthermore, we have determined potential causal mechanisms for this rectal injury, and published our findings on predictors of rectal tolerance to five fraction SBRT (2). Remarkably, even with the longest follow-up of the high-dose experience, no additional high grade rectal toxicity has developed in the 13 patients that were treated to 50 Gy in the phase I study. It appears that even with longer follow-up, the results from our phase I study would not have given us any additional indication that our MTD would have been anything <50 Gy for the phase II study.

**Table 2:** **Worst acute and delayed rectal toxicity in patients treated to 50 Gy dose in phase I and phase II studies**.

Rectal toxicity	All patients (*n* = 91)		50 Gy phase I (*n* = 14)		50 Gy phase II (*n* = 47)	
Grade	Acute No. (%)	Late No. (%)	Acute No. (%)	Late No. (%)	Acute No. (%)	Late No. (%)
0	39 (42.9)	38 (41.8)	5 (35.7)	5 (35.7)	18 (38.3)	15 (32)
1	33 (36.3)	27 (29.7)	7 (50)	6 (43)	16 (34)	15 (32)
2	17 (18.7)	21 (23.1)	2 (14.3)	2 (14.3)	11 (23.4)	13 (27.7)
3	1^a^ (1.1)	3 (3.3)	0 (0)	0 (0)	1^a^ (2.1)	3 (6.4)
4	1 (1.1)	2 (2.2)	0 (0)	1 (7.1)	1 (2.1)	1 (2.1)
Number of patients w/grade 3+ toxicity	6 (6.6%)		1 (7.1%)		5 (10.6%)	

*^a^For this patient, toxicity occurred on day 225 (acute period), but persisted to day 470, well into the delayed toxicity time period. Therefore, this patient was reported as having high grade acute and delayed toxicity. While seven total toxicities are reported, this occurred in a total of six patients*.

**Table 3:** **Stereotactic body radiation therapy studies**.

Study	N	Risk group (% of sample size)	Total dose (Gy)	Fraction size (Gy)	Median follow-up (months)	Biochemical control (%)	Grade 3+ GI toxicity
Katz (3)	304	LR 69	35–36.25	7–7.25	60	LR 97	0%
		IR 27				IR 90.7	
		HR 4				HR 74.1	
King (4)	1100	LR 58	35–40	7–8	36	LR 95	NR
		IR 30				IR 83	
		HR 11				HR 78	
McBride (5)	45	LR 100	36.25–37.5	7.25–7.5	44.5	97.7	5%
Oliai(6)	70	LR 51	35–37.5	7–7.5	27–33	LR 100	0%
		IR 31				IR 95	
		HR 17				HR 77.1	
Chen (7)	100	LR 37	35–36.25	7–7.25	27.6	99	0%
		IR 55	
		HR 8	
This study	91	LR 36	45–50	9–10	42	LR 100	6.6%
		IR 64				IR 98	
						All 99	
	30	LR 37	45–47.5	9–9.5	54	96.7	0%
		IR 63	
	61	LR 36	50	10	24.5	100	9.8%
		IR 64					

*LR, low risk; IR, intermediate risk; HR, high risk; GI, gastrointestinal*.

### Bowel quality of life outcome in the 50 Gy group: Phase I vs. phase II experience

**Figure 2:**
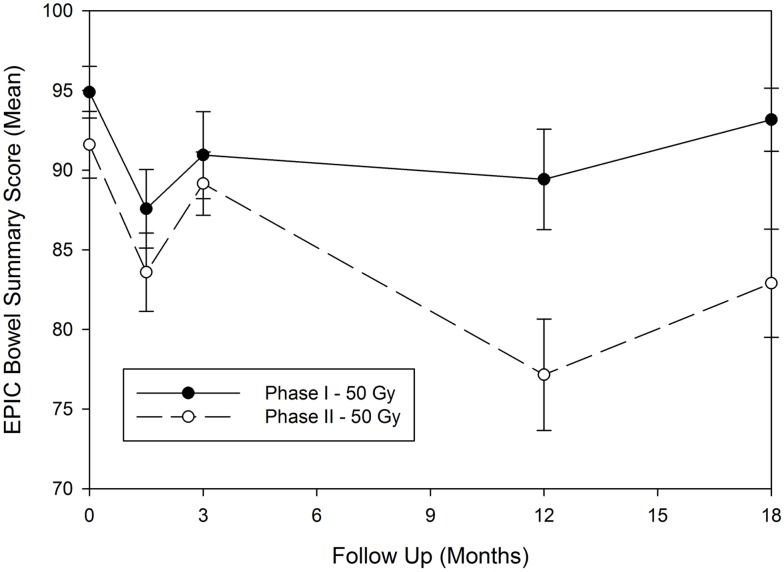
**Expanded prostate cancer index composite-bowel summary score for patients treated in the 50 Gy dose group for the phase I and phase II studies**. EPIC, expanded prostate cancer index composite; Gy, gray.

All patients in this study were required to undergo health related QOL questionnaires at baseline, and then at 1.5, 3, 12, and 18 months post SBRT. The EPIC questionnaires were used for this analysis. Given the discrepancy in the toxicity findings, we have further analyzed EPIC-Bowel symptom scores from patients who underwent 50 Gy in five fractions in the phase I (*n* = 14) and phase II (*n* = 47) study. Overall compliance to questionnaires was very good particularly early on in the study. Hundred percent and 96% of patients filled out baseline questionnaires in the phase I and II groups, respectively. By 18 months, data for EPIC-Bowel questionnaires were available from 78.5 to 53% in the phase I and II groups, respectively. As seen in Figure 2, the mean scores were fairly similar at 1.5 and 3 months post therapy with initial drop at 1.5 months followed by recovery at 3 months, reflective of acute toxicity events. However at 12 and 18 months, mean scores were lower in the patients treated in the phase II study, which may in part be due to the worse rectal toxicity noted in this group. In both groups, the EPIC-Bowel scores improved at 18 months compared to 12 months after treatment.

## Discussion

We have reported some of our interim findings from our phase I/II multi-institutional study of dose-escalated SBRT for localized prostate cancer. In the setting of a prospective, multi-institutional effort, we have treated patients starting at 45 Gy in five fractions to 50 Gy in five fractions, a dose that has been rarely reported in other series. Our prostate cancer control outcome in this study is excellent at 99% with a reasonable median f/u of 42 months. Of note, 53% of all patients in this study were GS 7, with 16.4% being GS 4 + 3. Interestingly, although we have only had one failure, this occurred in a patient who had GS 4 + 3 features. While we believe that his treatment failure was most likely attributable to regional biopsy proven nodal recurrence (3.4 years post therapy), it was intriguing to see that on prostate biopsy, there were concerns of two microscopic foci of residual prostatic adenocarcinoma 3.4 years post SBRT therapy even after 9 Gy × 5 fraction in this patient. While this pathologic data did not demonstrate a convincing evidence of local persistence of disease, it does give us a pause to consider that there may be certain aggressive subset of prostate tumors that can resist even doses as high as 9 Gy × 5 fractions, particularly for higher grade tumors. Interestingly, pooled analysis of SBRT experiences delivering median dose of 36.25 in 4–5 fractions suggested worse PSA outcome for intermediate and high risk disease with 5 years biochemical relapse free survival of 84 and 81%, respectively, compared to 95% for low risk patients (4). Similar findings were seen in study by Katz et al., with 5-year biochemical recurrence free survival reported to be 97, 90.7, and 74.1% for low, intermediate, and high risk patients, respectively (3). While some of these intermediate and high risk patients did receive doses as high as 38–40 Gy in five fractions (4), no published studies to date have reported doses in the order of 45–50 Gy as was the case in our study. Interestingly, in our study, patients treated with ≥47.5 Gy in five fractions, thus far have a 100% PSA control rate. This is the case even for patients in the 47.5 Gy group that have a respectable median f/u of 53 months (range 1.4–59.2 months). Of note, 7/15 patients in the 47.5 Gy group had GS 7 features, and 30/31 patients in the 50 Gy group had GS 7 features suggesting that local control was well maintained even in the intermediate risk patients using these higher doses.

However, our study, despite excellent PSA outcome, has demonstrated that there can be a risk of serious rectal injury at higher doses unless rectal constraints to limit vasculature/stromal destructive effects and to maintain a reasonable migration length to permit stem cell rescue of injured rectal mucosa are respected. We have specifically published on our findings that high grade rectal events were correlated with volume of rectal wall receiving 50 Gy > 3 cm^3^, and treatment of >35% of rectal wall to 39 Gy (2). What is remarkable from our phase I/II experience was that even when a respectable number of patients were treated in the 50 Gy of the phase I arm (*n* = 14), it required a larger sample size (*n* = 47) before it became apparent that we have reached a limit for rectal tolerance. This is reflected in the poorer QOL, and toxicity profile outcome (Table 2; Figure 2) in the patients treated in the phase II study vs. the 50 Gy arm of the phase I study. This is a sobering reminder to all of us that anecdotal experience of handful of patients should not suffice to give comfort when considering non-established treatments, and that properly designed prospective studies with adequate sample size are required when considering novel dose-escalated therapeutic approaches. What is apparent from our analysis was that such toxicity may be avoided if we follow the dose constraints discovered through this process (2), and that in the phase I study, 13 of the 14 patients happened to have met the dose constraints required to minimize high grade rectal events, most likely by chance.

Table 3 compares our experiences to some of the more contemporary studies with larger sample sizes that have been reported in the literature, including the consortium study (3–7). It is apparent that our outcome is comparable in terms of PSA control, all in support of the growing body of evidence that SBRT appears to be an effective treatment for low to intermediate risk prostate cancer. While studies do seem to support the use of 35–36.25 Gy for low risk patients, many of these are non-prospective studies. Our experience based on a rigorously designed and tested prospective trial, with a phase I dose-escalation scheme leading into the phase II study. Based on our findings thus far, we believe that 45–47.5 Gy is a safe and effective dose for further clinical studies. We do believe that longer follow-up would be required particularly for intermediate risk patients for all these studies including ours, to determine the potential merits or detriments of the higher doses used in our study. To further minimize rectal dose for future studies of SBRT, we are also exploring the use of an absorbable rectal spacer in conjunction with SBRT for our next studies involving low and intermediate risk patients (8). Furthermore, although additional long term follow-up data of patients treated at 45–47.5 Gy is warranted, given the paucity of further rectal issues on long term follow-up, and given the relative ease by which we can respect rectal dose constraints discovered from our analysis (2), we believe that these doses should prove to be safe, and possibly more potent and effective for patients with higher grade tumors. We are therefore using our experiences as a platform to develop clinical trials aimed at optimizing SBRT for patients with high risk prostate cancer who may require doses higher than currently reported in the literature (3, 4).

## Conflict of Interest Statement

The authors declare that the research was conducted in the absence of any commercial or financial relationships that could be construed as a potential conflict of interest.
